# Expansion of Electronic Health Record-Based Screening, Prevention, and Management of Diabetes in New York City

**DOI:** 10.5888/pcd10.120148

**Published:** 2013-01-31

**Authors:** Jeanine Albu, Nancy Sohler, Brenda Matti-Orozco, Jordan Sill, Daniel Baxter, Gary Burke, Edwin Young

**Affiliations:** Author Affiliations: Nancy Sohler, City College of New York, New York, New York; Brenda Matti-Orozco, Jordan Sill, Gary Burke, Edwin Young, St. Luke’s and Roosevelt Hospital Center, Columbia University, New York, New York; Daniel Baxter, the William F. Ryan Community Health Network, New York, New York.

## Abstract

To address the increasing burden of diabetes in New York City, we designed 2 electronic health records (EHRs)-facilitated diabetes management systems to be implemented in 6 primary care practices on the West Side of Manhattan, a standard system and an enhanced system. The standard system includes screening for diabetes. The enhanced system includes screening and ensures close patient follow-up; it applies principles of the chronic care model, including community–clinic linkages, to the management of patients newly diagnosed with diabetes and prediabetes through screening. We will stagger implementation of the enhanced system across the 6 clinics allowing comparison, through a quasi-experimental design (pre–post difference with a control group), of patients treated in the enhanced system with similar patients treated in the standard system. The findings could inform health system practices at multiple levels and influence the integration of community resources into routine diabetes care.

## Introduction

Significant progress has been made in controlling type 2 diabetes and its complications in primary care settings through the application of the chronic care model (CCM) (1–3). Evidence that CCM modifications to primary care practice can prevent type 2 diabetes is limited (4–6). Screening high-risk patients to detect diabetes and prediabetes was cost-effective (7–9), and prevention of type 2 diabetes in people with prediabetes through adoption of appropriate lifestyle changes and pharmacologic interventions has been successful in experimental settings (10). However, ongoing challenges in translating this evidence into primary care practice include the identification of appropriate target populations and the difficulty of incorporating time- and resource-intensive lifestyle interventions into routine clinical care (6). Although community and peer support systems have proven effective in preventing many chronic diseases (11,12), rigorous evaluations of integrated health care systems and community linkages for preventing type 2 diabetes are lacking (13).

Six clinics in a primary care network in New York City, 3 of which are federally qualified health centers, have established an evidence-based diabetes management system grounded in CCM principles in the context of developing a patient-centered medical home in each clinic (14). As defined by the National Committee for Quality Assurance, a patient-centered medical home is a health care setting that facilitates partnerships between patients and their physicians through the use of registries, information technology, and health information exchange. Our study will examine a standard and an enhanced diabetes management system. The standard system, already implemented, includes an electronic health records (EHR)-based, targeted screening program that is aimed at detecting previously undiagnosed diabetes (hemoglobin A1c [HbA1c] > 6.5%) and prediabetes (HbA1c 5.7%–6.4%). The enhanced diabetes management system is designed to facilitate the management of patients identified through the screening program as having diabetes or prediabetes. The enhanced system, which extends components of the CCM including community–clinic linkages to patient management, will be added to the standard system. The staggered implementation of the enhanced system across the 6 target clinics will allow comparisons of participant outcomes in the enhanced versus standard clinics through a quasi-experimental design (pre–post difference with a control group) by retrospective analyses of the data extracted from the EHR. The primary objective of these analyses is to test 3 hypotheses: first, that patients with newly diagnosed diabetes or prediabetes who will be exposed to the enhanced system will be more likely than patients exposed to the standard system to experience a reduction in HbA1c levels over 12 months; second, that any reductions in HbA1c levels observed in patients in the enhanced system will be sustained over a follow-up period of 30 months; and third, that patients in clinics adopting the enhanced system will be retained longer in appropriate health care than those in the standard system.

## Study Population

Patients seen in the 6 primary care clinics of the New York City health care system included in our study receive care that includes diabetes screening and management as determined by their health care provider. All patients who visit these clinics over a 6-month baseline period, are at least 18 years of age, and have not been previously diagnosed with diabetes are eligible for our study (15). Through an EHR-facilitated screening system (9,16,17), patients we identify as at risk for diabetes by American Diabetes Association (ADA) criteria (16) are referred for HbA1c tests. Those patients determined to have prediabetes (HbA1c 5.7–6.4%) or new-onset diabetes (HbA1c >6.5%) will be included in the study sample (15,16,18). All 6 clinics have patient populations with roughly similar sociodemographic and clinical characteristics.

## Study Design

All 6 clinics incorporated the standard diabetes management system over the first 6 months of 2012. Two clinics have been selected to start implementing the enhanced system within the following 6 months (from July through December 2012). For 12 months following implementation, these 2 clinics will use the enhanced system with all patients, while the remaining 4 clinics continue to use the standard system) until month 15 when they will also implement the enhanced system (Figure). We will follow all patients who meet study criteria and visit any of the 6 study clinics during the 6-month baseline period for a minimum of 30 months.

**Figure:**
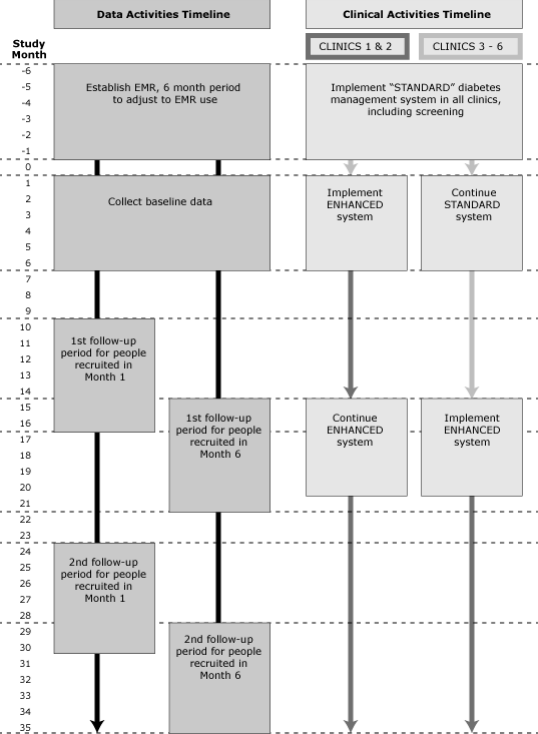
Data activities timeline and clinical activities timeline for standard diabetes management system and enhanced diabetes management system.

Follow-up care in the standard system conforms to recommendations for diabetes prevention and management (16); that is, patients with newly diagnosed diabetes are prescribed treatment and asked to return to the clinics every 3 to 6 months to monitor progress. We will advise patients newly diagnosed with prediabetes of their diagnosis; advise them of ADA-recommended lifestyle changes (5) via an office visit, a telephone call, or mail; and ask them to return within 12 months to assess progress in reducing HbA1c levels.

The enhanced system, in addition to the standard-system practices, fully incorporates CCM principles specific to the management of prediabetes. It addresses 1) care delivery and clinical information systems redesign (prediabetes-structured templates, planned visits, feedback on performance and point-of-care delivery prompts such as prediabetes order sets to ensure recommended clinical care); 2) decision support (education of physicians and ancillary staff in applying evidence-based prevention and treatment); and 3) patient self-management support (use of culturally appropriate tools, systematic referrals to established diabetes prevention programs in the community, and an inventory of available free or low-cost existing community resources that facilitate management of diabetes and prediabetes through lifestyle changes) (19,20). In the enhanced clinics, this self-management support system will also be available for patients with newly diagnosed diabetes. Patients with newly diagnosed prediabetes will be asked to return to the clinic within 6 months to monitor progress.

To ensure minimal losses to follow-up for our study, the staff in all 6 clinics will be instructed to contact all patients newly diagnosed with prediabetes or diabetes to ask them to return to clinic yearly.

## Data Analysis

We will conduct retrospective analyses on routine clinical data extracted from the EHR (Appendix). We will perform both intent-to-treat analysis and an as-treated analysis. Mixed-effects models accounting for clustering of patients within clinics will be used in our analyses. Our primary outcome, percentage change in HbA1c, will test our primary hypothesis that reduction in HbA1c observed at 12 months following initiation of the enhanced system in the selected clinics will be greater than reduction in HbA1c during the same period in the standard clinics. We will compare baseline HbA1c values to the average of HbA1c values obtained during the 12-month follow-up period. We will use this same outcome to test our second hypothesis, that observed HbA1c improvements in the enhanced clinics will be sustained over 30 months. To test our third hypothesis, that patients in clinics adopting the enhanced system will have greater retention in the health care system during the 12-month follow-up period than those exposed to the standard system, we will assess number of visits to a primary care physician and number of HbA1c tests performed. This will be a natural experiment; therefore, we anticipate some differences in the sociodemographic and clinical characteristics of participants across clinics. We will use propensity score-matching (21) to adjust for these differences, selecting from a wide array of patient measures in the EHR, including demographics, insurance coverage, clinical diagnoses, laboratory tests results, and information on use of health care services.

## Outcomes

This study will examine the effectiveness of an enhanced system for the management of newly diagnosed diabetes and prediabetes in participants identified through targeted screening in a primary care setting. This enhanced system will fully incorporate most components of the CCM (1,2) and as such, will involve policy changes at more than 1 level; however, its focus will be observation of the potential benefits of EHR in screening and monitoring outcomes and practice patterns (22). At the same time, this approach will involve a modest lifestyle change that leverages community-based resources. Evidence is mixed for the efficacy of EHR-based interventions in improving care for patients already diagnosed with diabetes (23,24). The use of EHR for diabetes screening and prevention may improve outcomes, because each primary care visit can be considered an opportunity for immediate intervention (25). If the outcomes of our analyses are positive, attribution of an EHR effect independent of other components of the intervention could be addressed in future studies.

Our study will be among the first to address whether it is possible to prevent diabetes in a large urban population by implementing an approach that is initiated during routine clinical practices and maintained over time by patients. A successful outcome could prompt health systems, health plans, and public insurers to adopt policies such as changes in reimbursement and reallocation of resources to facilitate prevention and early control of diabetes in primary care practices. In addition, it could prompt key players in the community (public agencies, business leaders, social organizations) to adopt and support policies aimed at developing and sustaining effective and affordable lifestyle intervention programs and at maintaining strong clinic–community linkages.

## APPENDIX. Power and Statistical Analyses

### Primary Analysis

Our primary outcome is defined as percent reduction in HbA1c. Baseline is the HbA1c measurement collected at the patient’s first visit, and we will obtain the baseline during the baseline period. This will be considered the pre-intervention measure. Final HbA1c will be the mean of all HbA1c measurements collected in the follow-up period. This will be considered the postintervention measure. This measurement must be made at least 12 months and no more than 24 months after the baseline measurement (Figure). The main “exposure” for our primary analysis will be receiving care from a provider in an enhanced diabetes management system site versus receiving care from a provider in a standard diabetes management system site. Other measures to adjust for potential noncomparability between groups include information on participant demographics, insurance coverage, clinical diagnoses, laboratory test results, use of laboratory services, and other measures of health services use collected on electronic medical records, including patient age, race, sex, zip code of residence, diabetes diagnosis, risk factor information, and other clinical data such as body mass index, blood pressure, blood lipids, and creatinine.

### Data Analysis

Our primary analysis will compare average change in participants’ HbA1c levels at the enhanced and standard sites during a 12-month period. For these analyses, the dependent variables will be baseline HbA1c and final HbA1c as defined above. Mixed-effects models accounting for clustering of patients within clinics will be constructed to test whether the average change in HbA1c is greater in the enhanced than in the standard system during a 12-month period. The models will include the following fixed effects: intervention type (eg, enhanced vs standard), time (baseline vs follow-up), and interaction effect (intervention by time). The models will include clinic and patient random effects to account for potential clustering among participants in the same clinic and between repeated measures in the same patient.

Because this is a natural experiment, we anticipate some differences in the sociodemographic and clinical characteristics of patients across clinics. We will use propensity score matching to adjust for these differences, selecting from a wide array of measures, which will be downloaded from our electronic health records (EHRs) to develop appropriate scores.

### Missing Data

We anticipate few missing data points because our data will draw from an EHR system. However, when missing data occur, we will examine these data for potential bias in missingness and will apply 1 of several possible imputation methods on the basis of our initial evaluations of the nature and extent of missing data. Because we anticipate any missing data will follow a multivariate normal distribution, we will first consider a standard Markov Chain Monte Carlo method, and we will impute missing data multiple (at least 5) times to take into account the uncertainty of imputed values. On the basis of the nature and form of missing data, we will also consider the other 2 widely used imputation methods, the predictive method and the propensity score method.

### Loss to Follow-up

In this design, loss to follow-up would include participants who drop out of care at the designated site (eg, move out of New York City), who do not keep any follow-up appointments within 12 months of an initial screening that yields positive results, or who do not have an HbA1c test.

### Power Analyses

Data from our EHR and published data from prior research (1) provided estimates for the following sample size calculations. These calculations were based on the method published by Heo and Leon (2). We applied the following outcome measure assumptions: 1) the log-transformed HbA1c measure (or other appropriate transformation) is approximately normally distributed; 2) the intraclass correlation of repeated HbA1c measures within a 12-month period is approximately 0.62; 3) the total population variance of HbA1c measures is 3.63; 4) the expected difference between the enhanced and standard systems in HbA1c change over 12 months is 0.3; and 5) there will be at least 2 clinics per exposure group. We will allow an α-error level of .05, and we anticipate a very conservative rate of loss to follow up of 15.0%. Given these assumptions, we have an 80.0% power to detect an average difference between the enhanced and standard group of 0.158 if we obtain a sample size of 568 patients assigned to each diabetes management system. Given the size of our health care system, we anticipate obtaining a sample size at least this large during our data collection period.

### Limitations

Because this is population-wide health services research, it is based on a nonrandomized design, leaving open the possibility of noncomparability between patients exposed to the enhanced system and those exposed to the standard system. Although we will consider this limitation as we conduct our analyses, biases and residual confounding are possible in this design. Participants at different practice sites are likely to differ in several ways. We will conduct subsequent analyses that examine each practice separately to make sure it is appropriate to combine these data. If not, separate results will be presented for each practice. However, this will reduce our power to detect a difference between the enhanced and standard systems.

### Secondary Analyses

In addition to the primary data analyses described above, we will conduct 2 secondary analyses.

1) We will examine whether observed improvements in the mean response profile of HbA1c of the enhanced diabetes management system is sustained after the first 12 months of follow-up. Because the enhanced system will be introduced to the 4 clinics using the standard system only at month 15, we will no longer have an unexposed concurrent control group. However, the available data will be useful for examining long-term trends over time for a large patient population. For example, we anticipate that the HbA1c outcomes for patients will continue to improve or remain stable over the longer-term observation period.

The measures and statistical models we will use for this analysis will be the same as those used for the primary analysis. In addition, we will consider using a combination of statistical techniques to compare the rate of change in participant HbA1c levels before and after implementation of the management systems, including lowess plots to assess the general shape of the curves, splining to account for nonpolynomial trends in the data, and linear mixed effects models. These findings will help us to draw conclusions about the overall effect of our diabetes management systems in our participant population.

2) We will also test whether patients in clinics adopting the enhanced system will be retained longer in the health care system and have greater adherence to recommended health care during the 12-month period following initiation of this system compared with those exposed to the standard system. For this analysis, our outcomes will include number of visits to a primary care physician, number of HbA1c measurements obtained, and number of community resources used. Prevention strategies can lead to unneeded medical care, that is, overuse of services. It will be important to monitor such unintended consequences and the cost of our enhanced diabetes management system. For example, we will closely monitor the number of HbA1c tests recorded for patients once their HbA1c levels are categorized as normal to determine whether unnecessary follow-up testing is being performed.

### References

1. Littenberg B, MacLean CD. Intra-cluster correlation coefficients in adults with diabetes in primary care practices: the Vermont Diabetes Information System field survey. BMC Med Res Methodol 2006;6 (20).

2. Heo M, Leon AC. Sample size requirements to detect an intervention by time interaction in longitudinal cluster randomized clinical trials. Stat Med 2009;28 (6):1017–27.
